# Association between selenium intake and cognitive function among older adults in the US: a critical analysis

**DOI:** 10.1017/jns.2023.86

**Published:** 2023-10-18

**Authors:** Janki Patel, Vrushank Shah, David F. Lo

**Affiliations:** 1 Lumina Institute of Research, Cream Ridge, NJ, USA; 2 Department of Biology, Rutgers, The State University of New Jersey, New Brunswick, NJ, USA; 3 Department of Medicine, Rowan University School of Osteopathic Medicine, Stratford, NJ, USA; 4 Department of Medicine, Rutgers Robert Wood Johnson Medical School, New Brunswick, NJ, USA

We read with pleasure the article by Ferdous *et al.*,^(1)^ titled ‘Association between selenium intake and cognitive function among older adults in the US: National Health and Nutrition Examination Surveys 2011–2014’ and would like to offer additional commentary on the extrapolated conclusions by assessing the impact of medications, diet, and viral infections on factors affecting selenium's bioavailability on cognitive function among older adults in the US. We hope these perspectives may provide insight that could be studied in future research endeavours.

First, the article does a wonderful job of providing insight into the manifold factors that influence oxidative stress, such as smoking status and alcohol intake. The study also eloquently highlighted selenium's effect on breaking down oxidants, and building upon this research will allow exploration of supplements and medications that may alter an individual's metabolism of oxidants, and impair cognitive function. For instance, cancer-related cognitive impairment (CRCI) has been observed in cancer patients during and after chemotherapy treatments from anthracyclines and platinum derivatives.^(2)^ Chemotherapeutic agents can directly damage DNA and generate oxidants, such as reactive oxygen species (ROS), as a by-product of DNA repair.^(3)^ Additionally, cancer drugs may dysregulate signalling pathways involved in maintaining antioxidant levels, such as MAPK and NF-κB. This generation of oxidants overwhelms the body's protective agents, leading to neuroinflammation and damage to neuronal structures as well as cognitive impairment.^(4)^ Moreover, psychoactive substances, such as amphetamines and cocaine, have been proven to generate metabolites and intermediates that cross the blood–brain barrier and cause an increase in ROS.^(5)^ These substances also shown to cause hyperphosphorylation of Tau proteins in the brain, which led to neuronal damage and cognitive impairment.^(6)^ Thus, further research is needed on cancer drugs as well as other psychoactive substances to help clarify the direct effects of selenium on oxidants and cognitive impairment, leading to further advancements in the field.

Second, the study mentioned the impact of selenium intake on its overall results, however, several studies have also shown that a diet high in antioxidants can greatly impact cognitive function.^(7)^ For example, the Mediterranean-DASH diet intervention for neurodegenerative delay (MIND) showed a positive correction in decreasing cognitive impairment, as it is known for its emphasis on fresh fruits, vegetables, whole grains, fish, etc., indicating significant results in improving cognitive function.^(8)^ Fish, such as salmon, mackerel, and sardines, are rich sources of omega-3 fatty acids, particularly docosahexaenoic acid (DHA) and eicosapentaenoic acid (EPA). These fatty acids are essential for brain health because they possess antioxidant properties and, therefore, neutralise free radical species.^(9)^ DHA and EPA also contribute to synaptic plasticity and neuronal communication by regulating neurotransmitters, thereby promoting memory and cognitive ability.^(10)^ Moreover, vegetables, such as spinach and other leafy greens contain beta-carotene, a fat-soluble vitamin that has antioxidant properties, while fruits in the MIND Diet contain flavonoids, which also protect against ROS.^(11)^ While the study accounted for several variables, including diet related to selenium intake, other aspects of diet are important to explore further, as a healthy diet could maintain, and potentially improve cognitive ability, skewing the statistical significance of selenium administration.

Third, though the study did a great job acknowledging many factors, such as cardiac and metabolic disorders that could skew the results, viral infections were not mentioned as a confounding variable, as infections are known to contribute to cognitive decline. Notably, about half of the individuals testing positive for human immunodeficiency virus (HIV) are affected by human immunodeficiency virus-associated neurocognitive disorder (HAND). HIV infects various cells and causes inflammation in the brain, ultimately causing cognitive decline.^(12)^ Additionally, COVID-19 is a viral infection and a fairly new area of research, which is currently being explored for impact on cognitive function. Although COVID-19 is primarily a respiratory illness caused by the SARS-CoV-2 virus, studies show that on average 7 months post-infection, patients show impairments in executive functions, attention, and memory due to unknown impacts on the central nervous system.^(13)^ Therefore, the research study must include individuals who have tested negative for specific viral conditions and are excluded from the study. At the same time, a newer study on this topic is necessary due to ongoing research on the SARS-CoV-2 virus on the nervous system and brain function.

The study did an excellent job taking into account many limitations; however, expansion of the study to different factors, such as medications and drugs, diet, and viral infections is necessary to elucidate selenium's impact on cognitive ability which would strengthen the validity of the study. In the end, we express our admiration to the authors for investigating the effects of selenium on cognitive impairment in the US, and we look forward to future studies providing insight into these factors.
